# Reward and aversion processing in patients with post-traumatic stress disorder: functional neuroimaging with visual and thermal stimuli

**DOI:** 10.1038/s41398-018-0292-6

**Published:** 2018-11-02

**Authors:** Igor Elman, Jaymin Upadhyay, Daniel D. Langleben, Mark Albanese, Lino Becerra, David Borsook

**Affiliations:** 1 Department of Psychiatry, Cooper Medical School, Rowan University, Glassboro, NJ USA; 2 Center for Pain and the Brain, Boston Children’s Hospital, Harvard Medical School, Boston, MA USA; 3 Department of Psychiatry, Perelman School of Medicine, University of Pennsylvania, Philadelphia, PA USA; 4 Cambridge Health Alliance, Harvard Medical School, Boston, MA USA

## Abstract

In patients with post-traumatic stress disorder (PTSD), a decrease in the brain reward function was reported in behavioral- and in neuroimaging studies. While pathophysiological mechanisms underlying this response are unclear, there are several lines of evidence suggesting over-recruitment of the brain reward regions by aversive stimuli rendering them unavailable to respond to reward-related content. The purpose of this study was to juxtapose brain responses to functional neuroimaging probes that reliably produce rewarding and aversive experiences in PTSD subjects and in healthy controls. The stimuli used were pleasant, aversive and neutral images selected from the International Affective Picture System (IAPS) along with pain-inducing heat applied to the dorsum of the left hand; all were administered during 3 T functional magnetic resonance imaging. Analyses of IAPS responses for the pleasant images revealed significantly decreased subjective ratings and brain activations in PTSD subjects that included striatum and medial prefrontal-, parietal- and temporal cortices. For the aversive images, decreased activations were observed in the amygdala and in the thalamus. PTSD and healthy subjects provided similar subjective ratings of thermal sensory thresholds and each of the temperatures. When 46 °C (hot) and 42 °C (neutral) temperatures were contrasted, voxelwise between-group comparison revealed greater activations in the striatum, amygdala, hippocampus and medial prefrontal cortex in the PTSD subjects. These latter findings were for the most part mirrored by the 44 vs. 42 °C contrast. Our data suggest different brain alterations patterns in PTSD, namely relatively diminished corticolimbic response to pleasant and aversive psychosocial stimuli in the face of exaggerated response to heat-related pain. The present findings support the hypothesis that brain sensitization to pain in PTSD may interfere with the processing of psychosocial stimuli whether they are of rewarding or aversive valence.

## Introduction

Reward deficiency, that is to say, hypofunctionality of the brain reward circuitry manifested in the diminution of drives and in inability to experience joy or pleasure^1^ is considered by some^2,3^ to be the most specific diagnostic^4^ feature of post-traumatic stress disorder (PTSD) documented in preclinical studies^5^ along with behavioral^6,7^ and neuroimaging^8,9^ clinical research. Although such neuropsychopathology is rather resistant to conventional therapies^10,11^ and is also associated with chronicity and severe disability^11,12^, its pathophysiological mechanisms remain poorly understood. One possibility is that reward hypo-responsivity is driven by an enduring brain alteration whether it be preexisting or acquired. A second possibility is that it is derived from a functional reciprocity between reward and stress reactivity^8^.

With regard to the former possibility, individuals afflicted with reward deficiency may perceive their life as bland and unfulfilling and possess a character trait of novelty seeking^13,14^, which could drive their engagement in stressogenic situations with an elevated potential for trauma exposure and subsequent PTSD^15,16^. This causality could run in the opposite direction^17^. That is to say, besides potent vasoconstriction^18^, chronic stress can exerts neurotoxic effects^19,20^ via a mix of related, but conceptually and operationally different mechanisms such as aggregation of platelets^21^, upsurge of intracellular calcium^22^ and acceleration of apoptosis^23^ evident in structural gray matter volume changes of the key corticolimbic structures^24,25^. Inherent in these structural changes are alterations in neural connectivity and/or neurochemisitry. For instance, reward deficiency is caused by dampening reward circuitry neurotransmission by way of enhanced dopamine metabolism^26^, its inhibited synthesis^27^ or extracellular release^28,29^ in conjunction with the reduction in dopamine receptors’ number^30^ and activity^31,32^.

It may as well be plausible that reward and stress alterations arising in the context of PTSD are temporally related owing to conspicuous neuroanatomical and functional overlap between the respective neurocircuitries^33,34^. Specifically, dopamine terminal fields, including amygdala, striatum and medial prefrontal cortex that are involved in the reward and motivational processing^35^ also play key roles in stress and aversion^36^. In patients with PTSD these areas^37,38^ become hypersensitive to trauma-conditioned environmental cues^8,39^, a mounting process leading to the generalization of fear^40,41^ that is added or synergized by the anti-reward cross-sensitization neuroadaptation amplifying responses to other aversive yet not necessarily conditioned stimuli^42–44^. Like so, in PTSD the same brain regions may be over-recruited by the aversive stimuli rendering them unavailable to respond to reward-related content and in the reversed order in people with low reward function aversive experiences (e.g., pain) are not buffered by reward and a consequence is the heightened pain experience^45,46^. These are testable hypothesis that could be evaluated by juxtaposing responses to functional neuroimaging probes that reliably produce rewarding and aversive experiences^8^.

Inquiry into aversion mechanisms in humans is limited in part by paucity of laboratory-based procedures that bring about strong and reproducible activation of major systems and that can be controlled with respect to the ‘amount’ of the administered stimulus. A paradigm well suited for examining aversive responses in humans is a common stressor^47^, experimentally-induced pain^48^. Consistent with the reward-aversion continuum conceptualization^49^, the brain’s pain system is embedded within extensive reward/motivation circuitry indispensable for the survival mechanisms via pursuit of nourishment while avoiding/escaping threats^44^. Even mild pain poses a sufficient aversive experience resulting in reliable brain and subjective responses^50^. Moreover, this procedure is not associated with performance confounds, so that equal ‘amounts’ of aversion are given to, both healthy subjects and to patients with a neuropsychiatric condition potentially entailing motivational^4^ and attentional^51^ deficits such as PTSD. Pain is also an ecologically valid stimulus to be used in PTSD patients as numerous epidemiological surveys indicate that the prevalence of chronic pain in PTSD patients exceeds that of the general population^52^ with up to a third of pain clinics’ patients afflicted with comorbid PTSD^53,54^ compared to a 4–12% PTSD rate in the general population^55^.

The purpose of the present study was to determine, employing functional magnetic resonance imaging (fMRI), whether PTSD is associated with primary vs. secondary alterations in reward processing. Two challenges used were (1) aversive or pleasant (i.e., rewarding) and neutral images^56^ selected from the International Affective Picture System (IAPS) and (2) pain-inducing noxious thermal stimuli^57^. The value of using these types of challenges is a more conclusive interpretation of the findings. Increased aversive stimuli (pain and negative IAPS images) responses in pain-free PTSD patients associated with signal decrements during rewarding (positive IAPS images) stimuli would support the notion that reward responsivity and pain sensitization are inversely related phenomena. Alternatively, if PTSD patients present the same directionality of the fMRI signal changes during both rewarding and aversive visual stimuli, it may be concluded that altered brain reward responses are not secondary to the over-recruitment of the brain reward regions by the aversive stimuli and a case for primary alterations in the brain reward and aversion function may be supported. Moreover, normal activity during pain, but not during aversive images’ processing, would suggest intact brain pain mechanisms and that fMRI signal differences are secondary to performance of the visual task. In a similar fashion, control level activity on both challenges would indicate that the respective brain circuitries are essentially intact with regard to their response to diverse rewarding and aversive challenges. Given that theoretical considerations on the above scores are not unambiguous directional prediction on rewarding vs. aversive stimuli responses was not sufficiently justified. Therefore, the hypothesis was formulated in terms of PTSD-related differences in the brain processing of both visual- and thermal-type of stimuli.

## Methods

### Subjects

Twelve subjects meeting the DSM-IV-TR criteria for PTSD, diagnosed via the Structured Clinical Interview for DSM-IV^58^ and Clinician-Administered PTSD Scale (CAPS)^59^, and 12 mentally healthy subjects were recruited by advertisement. After the procedures were fully explained, each subject gave written informed consent to the protocol approved by the McLean Hospital Institutional Review Board. All subjects were right-handed as assessed with Edinburgh Handedness Inventory;^60^ they were pain-free and in good physical health as determined by respective Brief Pain-^61^ and Cornell Medical Index Health Questionnaires^62^. Subjects with cognitive impairment or head trauma accompanied by amnesia or loss of consciousness greater than 10 min were excluded, as well as those with a history of schizophrenic-, paranoid-, other psychotic-, bipolar-, non-PTSD anxiety-, or substance dependence disorder. Given the high rate of depressive comorbidity in PTSD^63^, subjects with onset of major depressive disorder after the traumatic event that caused the PTSD were allowed to participate. Recent drug and alcohol consumption was ruled out by negative results on urine toxicology screen and breathalyzer. We also excluded the use within the previous month of any potentially confounding medications or drugs (e.g., opioids, psychostimulants, cannabinoids, dopaminergic or antidopaminergic agents, and mood stabilizers, antidepressants with prominent catecholaminergic effects such as tricylclics, buproprion, mirtazepine, venlafaxine, and duloxetine).

### Visual stimulation

Similar to our prior studies in mentally healthy subjects, emotional responses were probed using images selected from the IAPS^64^. Based on normative ratings for affective valance (unpleasant to pleasant) and arousal (calm to excited), three categories of images were selected: “pleasant”, “neutral”, and “aversive” categories. The pleasant images were the 90 pictures with the highest normative arousal scores selected from the 120 pictures with the highest normative valence intensity scores. Similarly, the aversive images were the 90 pictures with the highest normative arousal score selected from the 120 pictures with the lowest normative valence scores. Neutral images were 120 pictures with the highest normative arousal score selected from pictures with valence scores between 4.5 and 5.5 (range 1–9).

**Fig. 1:**
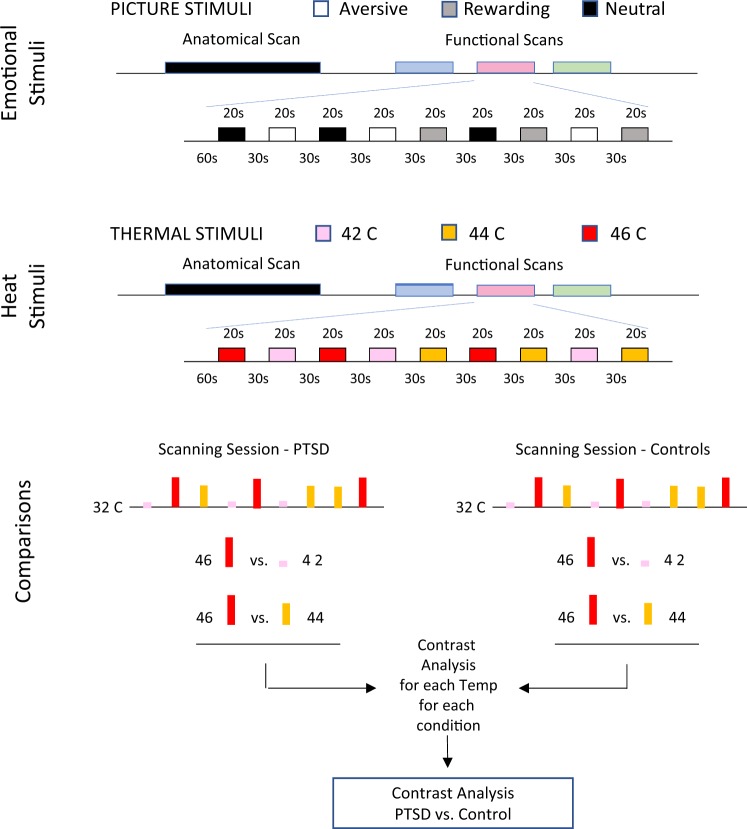
Imaging and data analytic protocol. Pain responses were probed by heat stimuli to the dorsum of the left hand delivered with a 3 × 3 cm contact thermode (TSA-II, Medoc Advanced Medical Systems). The thermode had a baseline temperature of 32 °C, and was rapidly heated (temperature rise = + 4 °C/s) to 42, 44 or 46 °C. The target temperature was maintained for 20 s and then returned to baseline (−4 °C/s) to end the stimulus event. Every subject received a total of nine thermal stimuli, three at each temperature, with an interstimulus interval of 30 s. To identify brain regions that differed between PTSD and healthy subjects, we contrasted 46 and 44 °C evoked responses to 42 °C. IAPS images data comparison was performed in an analogous fashion i.e., positive images minus neutral images and negative images minus neutral images. Both comparisons were calculated for each group (patients and controls) and for between group differences

IAPS images were presented in blocks of nine for each of the three categories (Fig. 1). Every subject had three fMRI scans, each with a total of nine visual stimulation blocks; three blocks of positive images, three blocks of aversive images and three blocks of neutral images. Each scan consisted of a 60 s baseline followed by nine visual stimulation blocks (20 s long) presented in pseudorandom order. Each image was only presented once. After each scan, subjects verbally rated the average valence experienced for the Pleasant and Aversive blocks using visual analog scale (VAS).

### Quantitative sensory testing

Prior to scanning, heat and cold thresholds were determined using a 3 × 3 cm contact thermode (TSA-II, Medoc Advanced Medical Systems). The temperature increased from a 32 °C baseline at the 1 °C/s rate until stopped by the subject at the first onset of pain. To determine cold pain thresholds, the skin was cooled down linearly at a slow rate (1 °C/s) until pain sensation was perceived (method of limits).

Similar to our prior studies in healthy subjects^65^, pain responses were probed by heat stimuli to the dorsum of the left hand delivered with a 3 × 3 cm contact thermode (TSA-II, Medoc Advanced Medical Systems). The thermode had a baseline temperature of 32 °C, and was rapidly heated (temperature rise = +4 °C/s) to 42, 44, or 46 °C. The target temperature was maintained for 20 s and then returned to baseline (–4 °C/s) to end the stimulus event. Every subject received a total of nine thermal stimuli, three at each temperature, with an inter-stimulus interval of 30 s. During each thermal stimulus, subjects rated pain intensity and unpleasantness using a rating dial in their right hand to adjust a VAS presented using the software package LabVIEW 5.1 (National Instruments Corp). Pain intensity was rated on a 0 to 10 VAS anchored at “No Pain” to “Max Pain”, unpleasantness was anchored at “Min” 0 to “Max” 10. To reduce expectancy confounds the stimuli were presented in a random order.

### Imaging protocol

A Siemens Trio 3 Tesla MRI scanner with a circularly polarized head coil was used for all scans. Brain structure was acquired with a magnetization prepared rapid gradient echo (MPRAGE) sequence [128 slices 1.33 mm thick, with an in-plane resolution of 1 mm (256 × 256)]. Blood-Oxygen-Level Dependent (BOLD) contrast functional scans were collected using an echo planar imaging sequence (echo time/repetition time (TE/TR) = 30/2500 ms for heat pain runs, TE/TR = 30/3000 ms for IAPS). The repetition times were optimized to the timing of the heat and visual probes. Both heat pain and visual functional scans consisted of 41 slices, with 3.5 mm isometric resolution. Eighty-four volumes were captured for each of the 42, 44 and 46 °C fMRI scans (3:30 each), and 199 volumes were captured for the IAPS fMRI scans (9:57). Visual and thermal stimuli were administered in a double blind counterbalanced fashion at least 15 min apart (Fig. 1).

### Data processing and voxelwise statistical analyses

Analysis was carried out using FSL tools release 5.0 (FMRIB Analysis Group, Oxford University; http://www.fmrib.ox.ac.uk/fsl/), specifically FEAT version number 5.92. Functional images were pre-processed using standard pipelines: motion correction, high pass temporal filtering (100 s), spatial smoothing (5 mm). Scans were inspected for gross motion with a threshold of 3 mm for elimination of the scan from further analysis. Images were registered to a standard atlas provided by FSL (MNI152 standard brain).

Statistical Analysis was carried out using a univariate general linear model approach; explanatory variables were created to represent the temporal presentation of thermal and visual stimuli. The resulting spatial parameter estimates were registered to standard atlas for group analysis. Group statistical analysis was carried out using a mixed-model approach as implemented in FSL; parameter estimate and variance images were included to perform the group comparisons described below. Inference was carried out using a Gaussian mixture model approach as described in ref. ^66^. Group and comparison statistical maps were subjected to alternative hypothesis testing without assuming normal distribution. The Gaussian mixture model approach produces posterior probability maps for the different classes of the original z-statistics map. Each voxel is associated with different classes with a specific (posterior) probability of belong to each class. Posterior probability maps were thresholded at 0.5 to determine brain regions statistically significant differences between the groups.

To search for brain regions that differed between PTSD and healthy subjects, we contrasted rewarding (pleasant) versus aversive (unpleasant) IAPS images (rewarding images minus neutral images and aversive images minus neutral images). Similarly, evoked responses to 46 and 44 °C were compared to 42 °C. Both comparisons were calculated for each group (patients and controls) and for between group differences. The t test results for each voxel were converted to z scores and thresholded to *p* < .01, at first uncorrected for multiple comparisons. All voxels with less significant activations (or deactivations of any magnitude) were excluded from further study. Remaining voxels were then collected into contiguous clusters. With Gaussian random field theory^67^, a significance level was associated with each cluster, this time correcting for multiple comparisons across the whole brain. Clusters with corrected significance at *z* > 2.3 and *p* < .05 were rendered as colored regions, with the color at each voxel indicating the corresponding z score.

The power analysis was based upon testing the IAPS response differences, which were likely to require more subjects consistent with a weaker response to psychosocial vs. physiological stimuli^68^. In our prior experiment with a psychosocial task^8^ the mean striatal BOLD signal changes in response to monetary reward in PTSD subjects was 0.05 ± 0.17 (SD) compared to 0.33 ± 0.35 in the healthy controls, yielding an effect size of 1.02d. We assumed the effect size for rewarding IAPS images to be comparably large. With 12 subjects in each group, we had 80% power at the *p* < 0.05 significance level to detect such an effect size for lower responses to reward in PTSD subjects.

## Results

### Demographic and clinical data

Table 1 presents demographic and clinical data for the study groups. These data demonstrate that PTSD subjects were not significantly different from healthy controls with respect to age, gender, years of education and performance on the quantitative sensory testing, but they scored significantly higher on the Harm Avoidance and Self-Transcendence and lower on Self-Directedness. The PTSD subjects also rated pleasant images significantly lower than healthy controls. As planned, there were conspicuous differences in the CAPS and Beck Depression Inventory-II^69^ scores.

**Table 1:** Demographic and clinical characteristics (mean ± standard deviation)

Characteristic	PTSD (*n* = 12)	Healthy (*n* = 12)
Age (year)	38.9 ± 11.9	39.6 ± 10.2
Gender (M/F)	5/7	6/6
Education (year)	14.9 ± 1.6	15.0 ± 2.4
**CAPS (score; range 0**–136)***	**76.5** **±** **13.5**	**0.8** **±** **2.6**
**BDI-2 (score; range 0**–63)***	**19.7** **±** **10.8**	**1.2** **±** **1.5**
Temperament and character inventory (score)		
Novelty seeking (range 0–40)	19.0 ± 6.3	16.3 ± 3.5
Harm avoidance** (range 0–**35)**	**20.5** **±** **8.4**	**10.6** **±** **4.9**
Reward dependence (range 0–24)	14.4 ± 3.7	17.3 ± 3.4
Persistence (range 0–8)	6.3 ± 1.1	5.4 ± 1.8
Self-directedness** (range 0–**44)**	**28.3** **±** **7.7**	**37.3** **±** **4.7**
Cooperativeness (range 0–42)	30.8 ± 8.6	37.6 ± 3.1
Self-transcendence* (range 0–**33)**	**16.6** **±** **6.6**	**11.3** **±** **5.9**
Self-ratings		
Quantitative sensory testing (threshold)		
Heat (°C)	44.2 ± 5.3	46.4 ± 3.9
Cold (°C)	11.9 ± 11.6	7.3 ± 6.8
46 °C unpleasantness (mm; range 0–10)	5.3 ± 4.0	6.3 ± 2.4
44 °C unpleasantness (mm; range 0–10)	3.2 ± 3.0	4.1 ± 2.1
IAPS pleasantness (mm; range 0–10)*	**6.1** **±** **1.3**	**7.2** **±** **1.0**
IAPS unpleasantness (mm; range 0–10)	2.2 ± 1.6	2.3 ± 1.1

*CAPS* Clinician-Administered PTSD Scale, *BDI-2* Beck Depression Inventory-II, *IAPS* International Affective Picture System

*p < 0.05, **p < 0.01, ***p < 0.001 (t-tests, independent by groups)

Significant group differences are bolded

### Imaging data

**Fig. 2:**
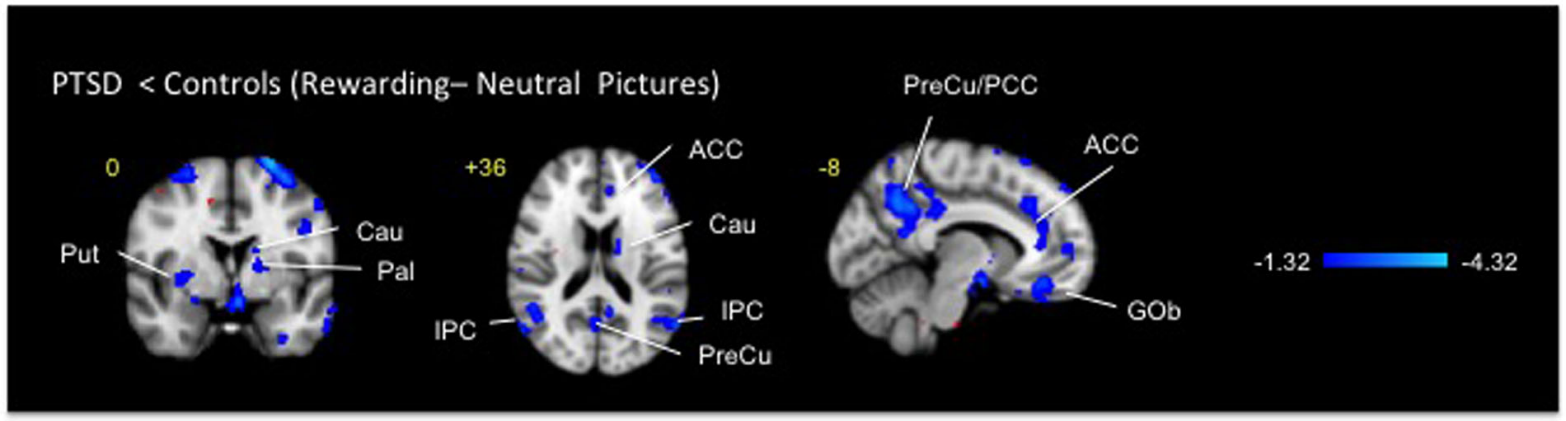
Clusters of deactivation obtained from voxelwise contrasts of IAPS positive-minus neutral images in PTSD and in healthy subjects (*n* = 12 in each group) projected onto a background (grayscale) representing subjects’ mean high-resolution anatomic image. Coordinates are in accordance with the Montreal Neurological Institute (MNI) space. ACC anterior cingulate cortex, Cau caudate, Gob orbitofrontal cortex, IPC inferior prefrontal cortex, Pal pallidum, PCC posterior cingulate cortex, PreCu precuneus, and Put putamen

**Fig. 3:**
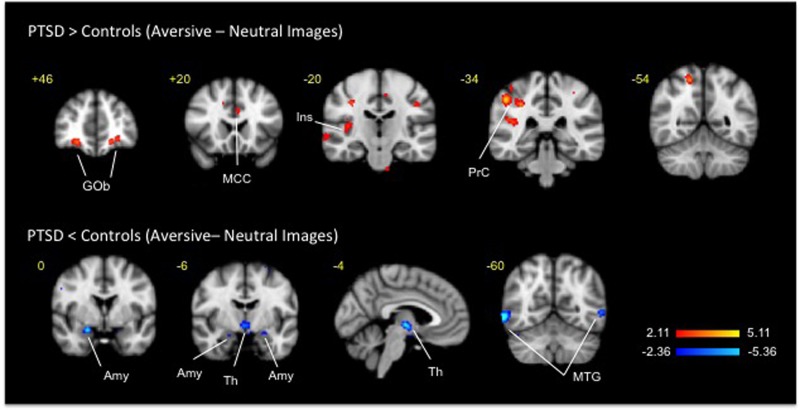
Clusters of activation and deactivation (respectively colored in red and blue) obtained from voxelwise contrasts of IAPS negative-minus neutral images in PTSD and in healthy subjects (*n* = 12 in each group) projected onto a background (grayscale) representing subjects’ mean high-resolution anatomic image. Coordinates are in accordance with the Montreal Neurological Institute (MNI) space. Amy amygdala, Gob orbitofrontal cortex, MCC midcingulate cortex, MTG middle temporal gyrus, Ins insula, PrC parietal cortex, Th thalamus

**Fig. 4:**
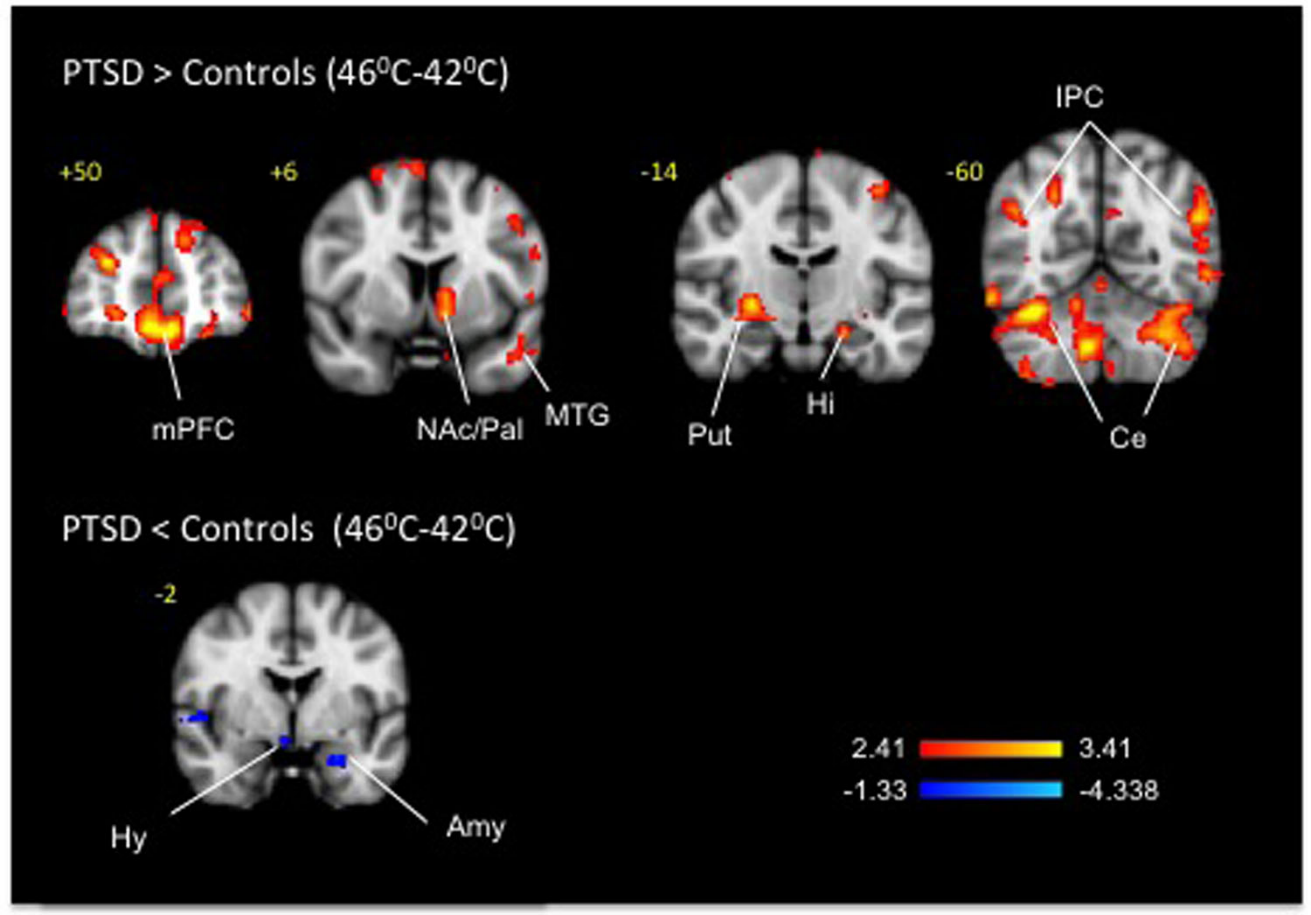
Clusters of activation and deactivation (respectively colored in red and blue) obtained from voxelwise contrasts of 44 °C-minus 42 °C in PTSD and in healthy subjects (*n* = 12 in each group) projected onto a background (grayscale) representing subjects’ mean high-resolution anatomic image. Coordinates are in accordance with the Montreal Neurological Institute (MNI) space. ACC anterior cingulate cortex, Amy amygdala, Cau Caudate, Ce cerebellum, Gob orbitofrontal cortex, Hi hippocampus, Hy hypothalamus, mPFC medial prefrontal cortex, MTG middle temporal gyrus, NAc nucleus accumbens, Pal Pallidum, Put putamen, Th thalamus, TP Temporal pole

**Fig. 5:**
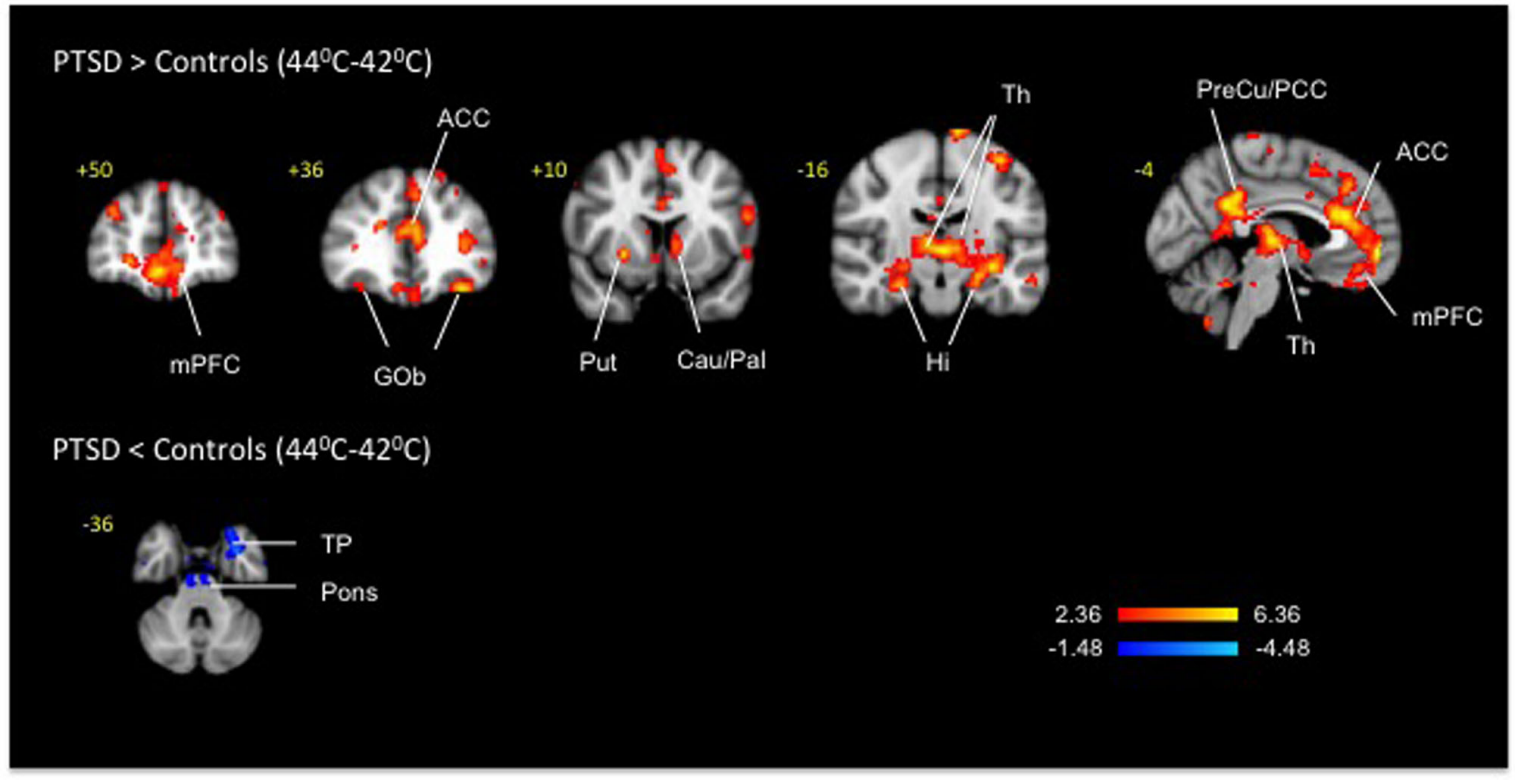
Clusters of activation and deactivation (respectively colored in red and blue) obtained from voxelwise contrasts of 46 °C-minus 42 °C in PTSD and in healthy subjects (*n* = 12 in each group) projected onto a background (grayscale) representing subjects’ mean high-resolution anatomic image. Coordinates are in accordance with the Montreal Neurological Institute (MNI) space. ACC anterior cingulate cortex, Amy amygala, Cau caudate, Ce cerebellum, Gob orbitofrontal cortex, Hi hippocampus, Hy hypothalamus, mPFC medial prefrontal cortex, MTG middle temporal gyrus, Pal pallidum, PreCu Precuneus, Put putamen

Imaging data are displayed in Figs. 2–5 and Tables 2–5 as regions within the brain divided into Cortical, Subcortical, and Brainstem/Cerebellum with *x*, y, and *z* coordinates in millimeters of the peak voxel and cluster volumes. Significant activations are noted in terms of z-statistics (z-stat). Because our prior work implicated striatum in hypofunctional reward responsivity in PTSD patients^8^, an *a priori* emphasis was placed on potential activations and deactivations in that area.

**Table 2:** Contrast analysis for rewarding IAPS stimuli (PTSD: IAPS positive–PTSD: IAPS neutral)–(Control: IAPS positive–Control: IAPS Neutral)

Brain region			Coordinates (mm)			Volume cm^3^
	Lat.	Zstat	*x*	*y*	*z*	
Negative						
Cortical						
Frontal						
Superior	R	2.0839	4	52	42	2.888
Rectus	L	1.5644	0	46	−18	0.464
Superior_Medial	L	2.1896	−2	40	56	0.416
Rectus	L	2.3799	−8	34	−18	1.96
Rectus	L	2.2608	0	22	−24	0.64
Precentral	L	3.5891	−54	10	32	26.856
Superior_Orbital	R	2.4536	26	2	64	1.464
Precentral	L	2.4556	−44	−2	32	0.592
Parietal						
Angular	R	1.7078	54	−50	32	0.376
Inferior	L	3.0534	−48	−52	56	12.472
Angular	R	2.6085	44	−52	36	1.36
Angular	R	3.3173	46	−58	24	5.32
Precuneus	L	2.898	−8	−60	38	8.624
Precuneus	L	1.8879	−4	−60	58	0.856
Occipital						
Calcarine	R	2.0683	4	−62	18	0.576
Temporal						
Pole_Middle	L	2.1849	−46	10	−32	1.344
Inferior	L	2.1111	−46	8	−36	0.896
Inferior	L	2.0172	−64	−34	−22	1.44
Inferior	R	2.1406	56	−48	−10	0.344
Middle	R	2.2183	56	−54	−2	0.544
Inferior	R	2.0425	64	−56	−4	0.416
Middle	L	3.1146	−60	−62	0	2.96
Cingulum						
Anterior	L	1.9482	−8	38	20	0.856
Middle	L	2.2833	−10	26	32	1.16
Parahippocampus						
Parahippocampal	R	1.6731	18	−4	−18	0.232
Sub-Cortical						
Putamen	R	2.215	28	2	2	1.096
Pallidum	L	1.8068	−20	0	6	0.224
Brainstem/Cerebellum						
Cerebellum_Crus2	R	2.254	40	−40	−42	0.456
Cerebellum_8	R	2.1839	36	−52	−50	0.352
Cerebellum_8	R	2.0246	24	−60	−48	0.456
Cerebellum_7b	R	2.5839	42	−64	−52	1.592

**Table 3:** Contrast analysis for aversive IAPS images (PTSD: IAPS Aversive–PTSD: IAPS Neutral)–(Control: IAPS Aversive–Control: IAPS Neutral)

Brain region			Coordinates (mm)			Volume cm^3^
	Lat.	Zstat	*x*	*y*	*z*	
Positive						
Cortical						
Frontal						
Superior_Orbital	R	2.56	34	54	10	0.456
Superior_Orbital	L	4.0537	−26	50	−8	0.968
Middle	L	3.2352	−24	50	8	0.928
Middle_Orbital	R	2.5548	28	40	26	0.224
Parietal						
Postcentral	R	2.6503	30	−26	56	0.336
Angular	L	2.5476	−44	−50	22	0.312
Occipital						
Rolandic_Operculum	R	2.8717	40	−14	18	0.296
Temporal						
Superior	R	2.7187	64	−16	0	1.744
Superior	R	2.8439	42	−36	18	0.272
Middle	L	2.4763	−50	−52	22	0.24
Brainstem/Cerebellum						
msn	R	2.8358	16	−30	−40	0.576
spV	L	2.8349	−2	−36	−60	0.456
Negative						
Cortical						
Frontal						
Inferior_Triangular	R	3.6038	50	28	14	0.664
Precentral	R	3.7138	62	8	20	0.736
Parietal						
SupraMarginal	L	3.5245	−64	−28	32	0.496
Temporal						
Inferior	R	4.4946	48	−50	−22	1.48
Inferior	L	3.7104	−54	−54	−20	0.752
Inferior	R	4.4622	60	−60	−4	2.976
Middle	L	3.9094	−56	−62	0	0.696
Sub-Cortical						
Amygdala	R	3.9166	22	0	−18	0.68
Thalamus	L	4.2201	−2	−12	−2	1.68

**Table 4:** Contrast analysis for pain i.e., Noxious Heat (PTSD: 46 °C–PTSD: 42 °C)–Control: 46 °C–Control: 42 °C)

Brain region	Lat.	Zstat	*x*	*y*		*z*	cm^3^
Positive							
Cortical							
Frontal							
Middle	R	4.5149	4	52		−12	6.216
Superior	L	3.3138	−14	50		34	2.912
Middle_Orbital	R	4.1583	28	48		22	5.256
Inferior_Triangular	R	3.088	50	46		−2	4.576
Superior_Medial	L	4.4573	−10	44		44	5.184
Middle	R	3.6398	6	44		−2	2.672
Inferior_Orbital	L	4.2308	−50	44		−8	6.816
Superior_Medial	L	3.2365	−8	42		24	1.888
Middle_Orbital	L	3.6153	−6	42		−14	0.88
Inferior_Orbital	L	3.3324	−34	38		−18	9.248
Rectus	L	3.8512	2	36		−24	4.568
Middle	L	3.0899	−30	34		42	2.344
Superior_Medial	L	3.6588	−6	32		40	2.976
Middle_Orbital	R	3.1365	40	32		22	11.888
Inferior_Triangular	L	3.2889	−50	32		14	3.848
Inferior_Orbital	R	3.6127	28	30		−24	2.392
Superior_Orbital	R	3.8527	14	28		52	3.488
Middle	L	3.1438	−50	20		40	2.84
Supp_Motor_Area	L	4.8875	−2	18		50	32.792
Precentral	L	3.1636	−46	2		40	9.48
Precentral	L	3.6611	−38	−16		58	9.592
Precentral	R	3.2171	32	−20		58	4.032
Parietal							
Angular	R	3.1444	48	−50		30	4.584
Inferior	L	3.7698	−54	−56		36	4.592
Angular	R	3.4722	46	−60		30	2.368
Angular	R	3.3966	44	−62		26	2.136
Angular	L	3.9151	−52	−62		26	3.192
Occipital							
Rolandic_Operculum	L	3.1518	−54	10		0	5.248
Temporal							
Middle	L	3.0839	−50	4		−22	1.664
Middle	L	4.1141	−42	2		−30	6.616
Inferior	L	3.1285	−38	−24		−30	10.24
Inferior	R	3.8777	48	−48		−8	6.768
Middle	L	3.4588	−44	−52		16	6.072
Inferior	R	3.2829	54	−52		−8	1.856
Middle	L	3.5635	−56	−60		−4	8.112
Inferior	R	3.7377	58	−60		−18	6.792
Cingulum							
Anterior	L	3.648	−6	42		8	6.88
Anterior	L	3.2944	−6	30		24	1.432
Anterior	L	4.1817	−6	22		22	5.728
Post	L	3.6965	−6	−48		28	8.08
Insula							
Insula_Anterior	R	3.1325	30	22		−16	1.992
Insula_Anterior	R	3.1263	36	4		16	3.96
Sub-Cortical							
Putamen	R	3.5437	26	14		0	4.256
Caudate	L	3.2604	−12	14		0	0.712
NAc	L	3.9158	−10	8		−6	4.928
Pallidum	L	3.4741	−14	2		−2	2.168
Amygdala	R	3.7746	32	−6		−12	5.96
Pallidum	R	3.9179	28	−14		−2	5.952
Hippocampus	L	3.7027	−22	−16		−16	7.408
Brainstem/Cerebellum							
PAG		3.179	−2	−26		−6	3.456
Cerebellum_8	L	3.1324	−22	−38		−50	1.432
Cerebellum_6	R	6.2048	36	−46		−26	21.496
Cerebellum_Crus2	L	3.5623	−42	−56		−42	5.088
Cerebellum_8	R	3.6938	34	−56		−54	7.368
Vermis_4_5		3.1742	0	−60		−10	7.072
Cerebellum_6	R	3.3647	14	−60		−20	2.216
Cerebellum_Crus1	L	4.0219	−32	−62		−34	2.904
Cerebellum_Crus1	L	4.0019	−36	−62		−34	1.92
Cerebellum_6	L	3.717	−36	−62		−24	3.816
Cerebellum_Crus1	R	3.7558	52	−64		−32	2.8
Negative							
Cortical							
Frontal							
Inferior_Orbital	L	2.1532	−30	34		−6	1.416
Rectus	L	2.9197	−6	26		−18	1.32
Middle_Orbital	R	2.0634	30	18		40	1.104
Precentral	L	1.9161	−36	0		30	0.336
Paracentral_Lobule	R	2.2028	8	−30		64	0.824
Parietal							
Postcentral	L	2.1779	−58	−10		40	0.816
Postcentral	R	2.4261	48	−18		40	1.344
Postcentral	R	2.7418	34	−30		40	1.192
Fusiform	R	2.0777	24	−30		−20	1.504
Postcentral	R	2.9956	22	−36		80	2.152
Postcentral	L	2.0547	−26	−40		78	1.624
Precuneus	L	2.0737	−16	−42		68	1.024
Precuneus	L	2.1096	−8	−44		78	0.424
Precuneus	R	2.3315	26	−50		2	0.968
Superior	R	2.0322	22	−50		70	0.424
Inferior	L	1.8085	−32	−52		48	0.256
Occipital							
Middle	L	2.1494	−28	−60		32	1.432
Temporal							
Superior	R	2.0631	62	2		−2	0.664
Superior	R	1.9796	66	−14		10	0.424
Superior	L	1.9296	−66	−26		6	0.456
Superior	R	2.3064	44	−42		4	1.152
Superior	R	1.856	62	−44		20	0.448
Lingual	R	2.0713	16	−46		−6	0.752
Middle	R	2.6184	64	−52		10	1.92
Cingulum							
Middle	R	2.8207	14	10		42	3.448
Middle	R	2.7638	12	−20		46	1.92
Insula							
Insula_Posterior	L	2.2091	−46	−10		4	0.616
Sub-Cortical							
Amygdala	L	2.2135	−24	−2		−22	0.48
Hypothalamus	R	2.2421	4	−4		−14	0.616
Brainstem/Cerebellum							
Cerebellum_Crus2	L	2.742	−52	−44		−42	1.112
Cerebellum_4_5	L	1.788	−8	−44		−4	0.232
Cerebellum_9	L	1.7861	−12	−52		−42	0.216

**Table 5:** Contrast analysis for mild heat (PTSD: 44 °C–PTSD: 42 °C) – (Control: 44 °C–Control: 42 °C)

Brain region			Coordinates (mm)			Volume cm^3^
	Lat.	Zstat	*x*	*y*	*z*	
Positive						
Cortical						
Frontal						
Middle	R	4.0704	6	48	−8	3.12
Middle_Orbital	R	3.2921	34	48	30	0.56
Superior	L	3.1293	−12	44	40	0.744
Rectus	L	3.2064	−2	42	−16	1.808
Rectus	R	3.7214	8	40	−18	1.056
Superior	L	3.4373	−14	40	56	0.944
Middle	L	3.4842	−38	40	22	1.368
Inferior_Triangular	L	3.5237	−34	36	8	1.568
Inferior_Orbital	L	3.8798	−34	36	−20	0.912
Superior_Medial	L	3.6091	−4	34	40	2.208
Middle	L	3.4024	−22	30	40	1.112
Inferior_Orbital	R	3.7141	28	30	−22	1.192
Inferior_Triangular	L	3.1173	−50	28	10	0.936
Inferior_Orbital	L	3.1268	−42	20	−4	0.872
Supp_Motor_Area	L	3.2645	−2	18	50	0.792
Supp_Motor_Area	L	3.4745	0	14	62	0.752
Inferior_Operculum	L	3.6119	−54	8	22	0.776
Precentral	L	3.4549	−54	4	26	0.44
Precentral	R	3.4352	54	−2	36	0.84
Precentral	L	3.4675	−52	−4	44	1.184
Superior_Orbital	R	3.2762	22	−8	64	0.32
Paracentral_Lobule	L	4.0229	−10	−14	78	1.16
Precentral	L	3.7143	−38	−16	60	1.84
Paracentral_Lobule	R	3.5266	2	−34	54	1.28
Parietal						
SupraMarginal	L	3.4122	−56	−32	26	0.584
Postcentral	L	3.1039	−30	−32	56	0.216
Postcentral	L	3.1249	−30	−36	60	1.376
Precuneus	L	3.5055	−6	−48	10	2.16
Inferior	L	3.1253	−38	−50	36	0.312
Precuneus	R	3.1816	16	−58	24	0.432
Precuneus	R	3.5186	14	−66	28	0.84
Precuneus	R	3.227	4	−66	26	0.888
Fusiform	R	3.7049	32	−66	−18	0.952
Occipital						
Rolandic_Operculum	R	3.2399	52	4	6	0.296
Temporal						
Middle	L	3.6741	−58	−12	−16	0.464
Superior	R	3.3562	58	−22	12	0.352
Lingual	L	3.6803	−14	−36	−4	1.4
Lingual	R	3.1735	10	−46	2	0.728
Inferior	R	3.5948	48	−48	−8	0.728
Middle	L	3.2165	−56	−50	−6	0.256
Middle	L	3.3457	−44	−52	16	0.76
Inferior	R	3.3425	52	−52	−8	0.656
Inferior	R	3.2641	48	−52	−12	0.248
Cingulum						
Anterior	L	3.3772	0	48	0	2.136
Anterior	L	3.6318	−4	38	14	0.664
Anterior	L	4.0637	−4	32	20	5.536
Anterior	L	3.1657	0	6	28	0.576
Post	R	3.2097	10	−36	10	0.976
Post	L	4.1616	−6	−48	28	4.648
Parahippocampus						
Parahippocampal	L	3.2434	−18	−40	−6	0.696
Sub-Cortical						
Caudate	L	3.2456	−12	14	0	1.072
Putamen	R	3.8053	24	10	−4	0.424
Hippocampus	R	3.593	26	−16	−20	1.176
Thalamus	L	4.299	−6	−22	10	12.704
Hippocampus	R	3.1028	18	−24	−10	0.28
Brainstem/Cerebellum						
Cerebellum_4_5	L	3.1111	−20	−38	−26	0.808
Cerebellum_6	R	3.3558	36	−46	−26	0.888
Cerebellum_9	R	3.2272	14	−48	−58	0.992
Cerebellum_8	R	3.5838	32	−54	−52	1.68
Cerebellum_9	R	4.1683	10	−56	−44	3.232
Cerebellum_8	R	3.3332	20	−58	−42	0.304
Cerebellum_6	R	3.6535	34	−66	−22	1.536
Negative						
Cortical						
Frontal						
Middle_Orbital	R	2.0443	40	30	30	0.32
Superior	R	2.4918	12	24	44	0.296
Inferior_Triangular	L	3.1483	−58	24	26	1.672
Middle_Orbital	R	1.8327	34	20	50	1.232
Superior	L	2.1947	−18	14	48	0.256
Middle_Orbital	R	2.6315	30	14	40	1.12
Parietal						
Postcentral	R	2.6851	32	−30	40	0.776
Postcentral	R	2.0626	38	−30	52	0.552
SupraMarginal	R	2.6399	50	−34	44	1.712
Postcentral	R	3.1791	46	−40	64	2.384
Inferior	R	2.471	40	−42	44	1.032
Inferior	R	2.1527	40	−50	54	0.416
Superior	R	3.5031	16	−56	56	0.96
Superior	R	2.2789	30	−56	56	0.56
Occipital						
Rolandic_Operculum	R	2.5939	66	12	10	0.432
Middle	L	1.956	−28	−60	32	0.376
Temporal						
Middle	R	1.9479	66	−52	10	0.256
Cingulum						
Middle	R	2.4938	14	22	38	0.48
Brainstem/Cerebellum						
msn	R	2.0307	8	−38	−48	0.272
Cerebellum_8	L	1.9706	−18	−62	−52	0.336

### Visual stimuli

For the processing of reward (Fig. 2, Table 2), between-group analyses of responses to the presentation of rewarding vs. neutral IAPS images in PTSD- vs. healthy subjects displayed 31 clusters of deactivation including cortical (cingulate, frontal occipital, parahippocampal, parietal and temporal), sub-cortical (right putamen and left pallidum), brainstem and cerebellum areas. Separate analyses in healthy and PTSD subjects revealed significant clusters of activations in the above regions for both groups; the clusters volumes and the level of significance were smaller in the PTSD group. For psychosocially aversive stimuli (Fig. 3, Table 3), between-group analyses of negative versus neutral IAPS images produced 12 clusters of activation (frontal occipital, parietal and temporal cortices and in cerebellum) and 9 clusters of deactivation (frontal parietal and temporal cortices, bilateral amygdala and thalamus). Separate analyses in healthy subjects detected large significant clusters of activation to negative minus neutral IAPS images that comprised bilateral frontal, temporal, occipital striatal and brainstem areas. Analyses in PTSD subjects observed small significant clusters of activation in bilateral temporal lobes and in thalamus.

### Thermal stimuli

Pair-wise group (PTSD-subjects vs. healthy subjects) comparison between noxious heat (46 °C) and mildly warm temperature (42 °C) (Fig. 4, Table 4) uncovered 60 clusters of activation in cortical (cingulate, frontal, hippocampal, occipital, parahippocampal, parietal, temporal and insular), sub-cortical (right amygdala, left caudate, left nucleus accumbens, right pallidum and right putamen), brainstem (periaqueductal gray) and cerebellar areas and 32 clusters of deactivation in the cortical (cingular, frontal, insular, occipital, parietal, temporal), sub-cortical (left amygdala and right hypothalamus) and cerebellar areas. In healthy subjects the 46 vs. 42 °C contrast resulted in a cluster of activation in the anterior cingulate and a bilateral cluster of deactivation in the hippocampus. When the PTSD group was considered in isolation, the 46 °C vs. 42 °C contrast detected bilateral activation clusters in the ventral and dorsal striatum comprised of nucleus accumbens and pallidum along with the clusters in the anterior cingulate and other cortical areas; deactivations were observed bilaterally in the hippocampus. Other than the prominent thalamic activations in the PTSD group that were not apparent in the 46 °C vs. 42 °C contrast potentially due to activation of the descending modulation system^70^, the 44 °C vs. 42 °C contrast (Fig. 5, Table 5) produced by and large a similar to the 46 °C vs. 42 °C contrast pattern of activations (62 clusters) and deactivations (20 clusters) in the cortical, subcortical and brainstem regions on both between groups and within group analyses.

## Discussion

To our knowledge, this is the first study to integrate reward and aversion subjective rating and neuroimaging data in patients with PTSD. The present results replicate others’^2^ and our earlier behavioral^6^, self-report^7^ and neuroimaging^8^ work uncovering PTSD-related decrements in response to rewarding visual stimuli and extend these prior findings by suggesting that, in addition to been numb to rewards, PTSD subjects may also be indifferent to some of the life’s discontents operationalized via aversive IAPS images as evidenced by bilateral deactivations in the key reward and aversion structure, amygdala. PTSD neuropsychopathology may thus encompass both positive and negative valence processing whether it is subserved by the same or by a different set of neurons^71^.

Decrease in cerebral metabolism and blood flow when exposed to natural reinforcers has been observed in a number of neuropsychiatric conditions (e.g., addiction and schizophrenia) characterized, like PTSD^8^, by diminished dopaminergic tone with corresponding decreases in the tonic glutamatergic activity due to drugs or to the disease process per se^35,72,73^. On the background of this diminished activity, respective exposure to drugs, to conditioned cues or to psychotic contents leads to robust augmentations of phasic corticolimbic responses^72^ akin to pain-induced activations on the present study.

A prior neuroimaging investigation with the laboratory-based pain induction found greater activations in hippocampus, putamen and insula and less activations in the amygdala and prefrontal cortex of combat PTSD Veterans during their exposure to a fixed and customized (to subjective ratings) temperatures^74^. That study did not, however, obtain baseline pain assessments and subjective pain thresholds. A subsequent study in women only replicated the insular activations finding^75^. The direction of PTSD subjects’ subjective responses to experimentally-induced pain varied and resting state hyperalgesia^76,77^, hypoalgesia^74,76,78^ and no differences^79^ when compared to healthy subjects have been reported. Methodological factors^80^ such as inter-subject pain threshold variability^74,76^, individualized vs. standardized magnitude of the pain stimuli^74^, concurrent PTSD symptoms reactivation^79^, pain expectancy context^81^ and presence of comorbid pain conditions^76^ may explain the divergent pain effects in PTSD.

It has been previously suggested that PTSD patients are not actually numb and that their capacity to experience positive emotions is rather constrained by preferential allocation of emotional, motivational and cognitive resources to environmental threats including re-experiencing of the traumatic episodes^82,83^. Partially overlapping hypo and hyper in the PTSD subjects (e.g., left pallidum^84^) respectively produced by the positive IAPS images and by pain supports the possibility that PTSD patients deactivate and activate the same brain structures to respective rewarding and aversive stimuli. However, even if such structures are identified in this and prior functional and/or structural neuroimaging studies, the microcircuits located within those structures may actually carry out discrete and non-overlapping tasks. Emerging neuroscience technologies integrating viral vectors with optogenetics in combination with in vivo single cell recording, electrophysiology and neuroanatomical analyses^85,86^ afford higher (than human neuroimaging) resolution of neural underpinning of normal function and of pathopysiological processes. The present findings thus provide a foundation for preclinical studies applying concurrent reward and stress measurements in PTSD models to further address the questions of reward and stress circuitries’ interactions.

We also observed dissociation between brain activations and quantified measures of pain valuation. Specifically, pain free subjects with PTSD rated painful stimuli similarly to healthy controls, but displayed greater brain activations to the same stimuli. This group difference was not explained by variability of pain thresholds. Such heightened brain pain responses notwithstanding regular self-reports may point to enhanced brain’s ability to screen out/suppress responses to seemingly irrelevant^87^ noxious and other types of stimuli from reaching conscious awareness^88,89^ i.e., “gating”^87^. While disrupted sensorimotor gating plays an important role in the course of PTSD^90^, the present finding of similar unpleasantness ratings of the aversive IAPS images in the face of decreased activations in the PTSD group renders enhanced gating an unlikely mechanism of the observed dissociations between neuroimaging findings and subjective ratings. Nonetheless, electroencephalography^91^ and magnetoencephalography^92^ could be used in conjunction with pain probes to examine further questions concerning sensorimotor gating mechanisms underlying PTSD symptomatology.

Another issue to consider is the cross-sensitization phenomena^44,93^. This term pertains to a situation where prior exposure to one stimulus (e.g., trauma and its consequent re-experiencing) increases subsequent response to itself and to a different stimulus (e.g., pain). The cross-sensitization did not seem to include brain responses to another aversive stimulus used on the study, i.e., negative IAPS images, which may have been attenuated because of a possible ‘floor effect’ given the low subjective ratings. Emotional processing may be attributed to a two–system construct^94^ comprised of corticolimbic circuits mediating valence (ranging from aversive to rewarding) in conjunction with closely linked networks coding intensity-related arousal^71^. Future research may consider matching negative stimuli by the level of intensity to address the generalizability of the cross-sensitization processes.

The mechanisms of cross-sensitization may involve conditioning. Thus, pain, paired with emotional trauma and its recollections, can become a conditioned stimulus that evokes fear and anxiety responses that in turn augment subjective pain perception and its neural correlates^95,96^, and so mounts the “mutual maintenance”^97^ cycle, leading to additional deterioration and avoidance of pain- and trauma-related situations^98–101^. Formulation of PTSD treatment plans targeting emotional numbing might then benefit from the habituation and extinction of stressful re-experience techniques^102^ along with provision of potent positive stimuli^83^.

In addition, increased central opiodergic tone^79,103^ along with robust elevations of endogenous opiates concentrations in the cerebral spinal fluid^104^ and in plasma^105,106^ is a relatively consistent clinical finding in PTSD. Therefore, similarly to chronic users of opioid pain relievers^107,108^, PTSD-related exaggerated CNS opioidergic activity could contribute to sensitized brain pain responses mediated via the amplification of the excitatory (e.g., glutamtergic) neurotransmission^109–111^. If such neurobiologic vulnerability factors could be identified, they might be used to screen patients at risk for the development of pain condition. Patients found to possess high vulnerability for the development of pain owing to PTSD-related heightened opioidergic tone function might be counseled to avoid opioids (primary prevention), or targeted for early intervention with non-opioid agents^49^ even in the presence of mild pain problems (secondary prevention).

Yet, in order to prevent sensitization of the healthy brain aggressive and timely analgesic treatment may actually be indicated. In fact, peritraumatic pain is a stressor recognized than an independent PTSD risk factor^112^ whereas chronic pain may be construed as a variant of PTSD due to persistent relieving of stress, avoidance of pain-related situations and negative cognitions and affective states^44^. This may be why adequate morphine^113,114^ or ketamine^115^ analgesia reduces the severity and may even prevent the appearance of PTSD. An additional therapeutic implication of the opioidergic mechanisms’ involvement in PTSD pathophysiology^79,116,117^ is the clinical use of opioid antagonists^103,118,119^ in some pain-free patients, that on the whole appears to be safe and well tolerated and results in significant improvements of various aspects of PTSD symptomatology such as emotional numbing, startle response, nightmares, flashbacks, intrusive thoughts and comorbid alcoholism^103,118,120^.

### Caveats

Caveats that should be considered in interpreting our data refer to the type of stimuli, the duration of the study and the pilot nature of the study design. First, although the aversive state created by the thermal stimuli is qualitatively different from environmentally-induced pain that is implicated in PTSD pathophysiology^112^, we believe that our results may have clinical significance because real life pain affects similar brain areas to those produced by the heated thermode^121^. Likewise, both aversive stimuli employed may have been quantitatively different from environmental stressors that have been implicated in initiation and exacerbation of PTSD. While we involved both psychosocial and sensory components, the subjective ratings of averseness were only moderately affected. Because various stressors may have diverse effects on regulatory systems, future studies employing other types of aversive stimuli than the ones previously used by our group e.g., glucoprivation with 2-deoxyglucose^122^ or adrenergic stimulation with yohimbine^123^ may provide unique information pertaining to general stress and corticolimbic responsiveness. Also, even though we employed visual and sensory stimuli of aversive quality they engage different behavioral and emotional systems the overlap of which may not necessary be aversion processing per se. This systems’ parameter can be isolated by comparing brain response in subjects who do experience versus who do not experience aversion from the presented stimuli.

Second, the observed group differences in reward processing may reflect a pre-existing risk factor rather than an acquired neuropsychopathology resulting from trauma exposure and subsequent PTSD. If this were the case, the PTSD subjects would have displayed purportedly heritable personality traits that are suggestive of the reward deficiency^124^. The Temperament and Character Inventory’s Novelty Seeking and Reward Dependence data render this option unlikely and suggest that premorbid reward function in PTSD subjects was similar to that in the control group. Yet, the effects of premorbid factors particularly related to Harm Avoidance, Self-Directedness and Self-Transcendence that differentiated PTSD and control groups on this study is an important consideration for the future research regarding the origin of PTSD-related reward deficits. Third, this study assessed only acute pain response while evidence suggests that such response tend to sensitize over time^125^. Therefore, longer study periods may have yielded different results. Finally, these findings should be considered as preliminary pending replication with a larger sample.

## Conclusions

In conclusion, pilot data presented here suggest that reward and pain activate partially overlapping corticolimbic areas. Patients with PTSD display reward hypo-responsivity notwithstanding excessive responses to pain. At the same time, subjective group differences in response to aversive psychosocial images are not obvious. These data shed light on pathophysiology of reward and aversion disturbances to suggest their reciprocity in PTSD and call for further research aimed at understanding the distinctive features of reward vis-à-vis pain alterations and their potential role in preventive efforts and in therapeutic armamentarium for the respective patients.

## References

[1] Blum K (2000). Reward deficiency syndrome: a biogenetic model for the diagnosis and treatment of impulsive, addictive, and compulsive behaviors. J. Psychoact. Drugs.

[2] Nawijn L (2015). Reward functioning in PTSD: a systematic review exploring the mechanisms underlying anhedonia. Neurosci. Biobehav. Rev..

[3] North CS, Suris AM, Davis M, Smith RP (2009). Toward validation of the diagnosis of posttraumatic stress disorder. Am. J. Psychiatry.

[4] American Psychiatric Association Publishing. Diagnostic and Statistical Manual of Mental Disorders, 5th edn. (Washington, DC, 2013).

[5] Nestler EJ (2015). Role of the Brain’s Reward Circuitry in Depression: Transcriptional Mechanisms. Int. Rev. Neurobiol..

[6] Elman I (2005). Probing reward function in post-traumatic stress disorder with beautiful facial images. Psychiatry Res..

[7] Hopper JW (2008). Probing reward function in posttraumatic stress disorder: expectancy and satisfaction with monetary gains and losses. J. Psychiatr. Res..

[8] Elman I (2009). Functional neuroimaging of reward circuitry responsivity to monetary gains and losses in posttraumatic stress disorder. Biol. Psychiatry.

[9] Frewen PA (2012). Emotional numbing in posttraumatic stress disorder: a functional magnetic resonance imaging study. J. Clin. Psychiatry.

[10] Taylor S (2001). Posttraumatic stress disorder arising after road traffic collisions: patterns of response to cognitive-behavior therapy. J. Consult. Clin. Psychol..

[11] Hassija CM, Jakupcak M, Gray MJ (2012). Numbing and dysphoria symptoms of posttraumatic stress disorder among Iraq and Afghanistan War veterans: a review of findings and implications for treatment. Behav. Modif..

[12] Hassija CM, Luterek JA, Naragon-Gainey K, Moore SA, Simpson T (2012). Impact of emotional approach coping and hope on PTSD and depression symptoms in a trauma exposed sample of Veterans receiving outpatient VA mental health care services. Anxiety Stress. Coping..

[13] Bardo MT, Donohew RL, Harrington NG (1996). Psychobiology of novelty seeking and drug seeking behavior. Behav. Brain. Res..

[14] Dellu F, Piazza PV, Mayo W, Le Moal M, Simon H (1996). Novelty-seeking in rats--biobehavioral characteristics and possible relationship with the sensation-seeking trait in man. Neuropsychobiology.

[15] Richman H, Frueh BC (1997). Personality and PTSD II: personality assessment of PTSD-diagnosed Vietnam veterans using the cloninger tridimensional personality questionnaire (TPQ). Depress Anxiety.

[16] Shumake J, Barrett D, Gonzalez-Lima F (2005). Behavioral characteristics of rats predisposed to learned helplessness: reduced reward sensitivity, increased novelty seeking, and persistent fear memories. Behav. Brain. Res..

[17] Der-Avakian A, Mazei-Robison MS, Kesby JP, Nestler EJ, Markou A (2014). Enduring deficits in brain reward function after chronic social defeat in rats: susceptibility, resilience, and antidepressant response. Biol. Psychiatry.

[18] McEwen BS (2007). Physiology and neurobiology of stress and adaptation: central role of the brain. Physiol. Rev..

[19] Vachon-Presseau E (2017). Effects of stress on the corticolimbic system: implications for chronic pain. Prog. Neuro-psychopharmacol. Biol. Psychiatry.

[20] Arnsten AF, Raskind MA, Taylor FB, Connor DF (2015). The Effects of Stress Exposure on Prefrontal Cortex: Translating Basic Research into Successful Treatments for Post-Traumatic Stress Disorder. Neurobiol. Stress.

[21] Stämpfli SF (2014). Restraint stress enhances arterial thrombosis in vivo--role of the sympathetic nervous system. Stress.

[22] Anguita E, Villalobo A (2018). Ca2+signaling and Src-kinases-controlled cellular functions. Arch. Biochem. Biophys..

[23] Li Y, Han F, Shi Y (2013). Increased neuronal apoptosis in medial prefrontal cortex is accompanied with changes of Bcl-2 and Bax in a rat model of post-traumatic stress disorder. J. Mol. Neurosci..

[24] Zach P (2016). Effect of stress on structural brain asymmetry. Neuro. Endocrinol. Lett..

[25] Gold MS (2018). Molecular role of dopamine in anhedonia linked to reward deficiency syndrome (RDS) and anti- reward systems. Front. Biosci..

[26] Dalla C (2008). Sex differences in the effects of two stress paradigms on dopaminergic neurotransmission. Physiol. Behav..

[27] Czyrak A, Mackowiak M, Chocyk A, Fijal K, Wedzony K (2003). Role of glucocorticoids in the regulation of dopaminergic neurotransmission. Pol. J. Pharmacol..

[28] Tidey JW, Miczek KA (1996). Social defeat stress selectively alters mesocorticolimbic dopamine release: an in vivo microdialysis study. Brain Res..

[29] Gambarana C (1999). A chronic stress that impairs reactivity in rats also decreases dopaminergic transmission in the nucleus accumbens: a microdialysis study. J. Neurochem..

[30] Zeman P, Alexandrova M, Kvetnansky R (1988). Opioid mu and delta and dopamine receptor number changes in rat striatum during stress. Endocrinol. Exp..

[31] Zhu X, Peng S, Zhang S, Zhang X (2011). Stress-induced depressive behaviors are correlated with Par-4 and DRD2 expression in rat striatum. Behav. Brain. Res..

[32] Moriam S, Sobhani ME (2013). Epigenetic effect of chronic stress on dopamine signaling and depression. Genet. & epigenetics.

[33] Zacharko RM, Anisman H (1991). Stressor-induced anhedonia in the mesocorticolimbic system. Neurosci. Biobehav. Rev..

[34] Puglisi-Allegra S, Imperato A, Angelucci L, Cabib S (1991). Acute stress induces time-dependent responses in dopamine mesolimbic system. Brain Res..

[35] Koob GF, Volkow ND (2016). Neurobiology of addiction: a neurocircuitry analysis. Lancet Psychiatry.

[36] Dunn AJ (1988). Stress-related activation of cerebral dopaminergic systems. Ann. N. Y. Acad. Sci..

[37] Doronbekov TK (2005). Neural basis of fear conditioning induced by video clip: positron emission tomography study. Psychiatry Clin. Neurosci..

[38] Liberzon I (1999). Brain activation in PTSD in response to trauma-related stimuli. Biol. Psychiatry.

[39] Liberzon I, Sripada CS (2008). The functional neuroanatomy of PTSD: a critical review. Prog. Brain Res..

[40] Ross DA (2017). An Integrated Neuroscience Perspective on Formulation and Treatment Planning for Posttraumatic Stress Disorder: An Educational Review. JAMA Psychiatry.

[41] Lopresto D, Schipper P, Homberg JR (2016). Neural circuits and mechanisms involved in fear generalization: Implications for the pathophysiology and treatment of posttraumatic stress disorder. Neurosci. Biobehav. Rev..

[42] Robinson TE, Berridge KC (2003). Addiction. Annu. Rev. Psychol..

[43] Borsook D (2016). Reward deficiency and anti-reward in pain chronification. Neurosci. Biobehav. Rev..

[44] Elman I, Borsook D (2016). Common Brain Mechanisms of Chronic Pain and Addiction. Neuron.

[45] Sadowski B, Marek P, Panocka I (1984). Enhancement of performance for brain stimulation reward after footshock in rats. Acta Neurobiol. Exp. (Wars.).

[46] Fields HL, Hjelmstad GO, Margolis EB, Nicola SM (2007). Ventral tegmental area neurons in learned appetitive behavior and positive reinforcement. Annu. Rev. Neurosci..

[47] Chapman CR, Tuckett RP, Song CW (2008). Pain and stress in a systems perspective: reciprocal neural, endocrine, and immune interactions. J. Pain..

[48] Quiton RL, Keaser ML, Zhuo J, Gullapalli RP, Greenspan JD (2014). Intersession reliability of fMRI activation for heat pain and motor tasks. NeuroImage Clin..

[49] Elman I, Zubieta JK, Borsook D (2011). The missing p in psychiatric training: why it is important to teach pain to psychiatrists. Arch. Gen. Psychiatry.

[50] Moeller-Bertram T, Keltner J, Strigo IA (2012). Pain and post traumatic stress disorder - review of clinical and experimental evidence. Neuropharmacology.

[51] Block SR, Liberzon I (2016). Attentional processes in posttraumatic stress disorder and the associated changes in neural functioning. Exp. Neurol..

[52] Weisberg RB (2002). Nonpsychiatric illness among primary care patients with trauma histories and posttraumatic stress disorder. Psychiatr. Serv..

[53] Beckham JC (1997). Chronic posttraumatic stress disorder and chronic pain in Vietnam combat veterans. J. Psychosom. Res..

[54] McWilliams LA, Cox BJ, Enns MW (2003). Mood and anxiety disorders associated with chronic pain: an examination in a nationally representative sample. Pain.

[55] Kessler RC (2005). Lifetime prevalence and age-of-onset distributions of DSM-IV disorders in the National Comorbidity Survey Replication. Arch. Gen. Psychiatry.

[56] Wilcox SL (2016). Increased Functional Activation of Limbic Brain Regions during Negative Emotional Processing in Migraine. Frontiers in Human. Neuroscience.

[57] Upadhyay J (2015). Test-retest reliability of evoked heat stimulation BOLD fMRI. J. Neurosci. Methods.

[58] First M, Spitzer RL, Gibbon M, Williams JBW (1995). Structured Clinical Interview for DSM-Iv Axis I Disorders - Patient Edition (SCID-I/P, Version2.0).

[59] Weathers FW, Keane TM, Davidson JR (2001). Clinician-administered PTSD scale: a review of the first ten years of research. Depress Anxiety.

[60] Oldfield RC (1971). The assessment and analysis of handedness: the Edinburgh inventory. Neuropsychologia.

[61] Atkinson TM (2010). The Brief Pain Inventory and its “pain at its worst in the last 24h” item: clinical trial endpoint considerations. Pain. Med..

[62] Seymour GE (1976). The structure and predictive ability of the Cornell Medical Index for a normal sample. J. Psychosom. Res..

[63] Kessler RC, Sonnega A, Bromet E, Hughes M, Nelson CB (1995). Posttraumatic stress disorder in the National Comorbidity Survey. Arch. Gen. Psychiatry.

[64] Lang, P. J., Bradley, M. M., Cuthbert, B. N. *International affective picture system (IAPS): Affective ratings of pictures and instruction manual*. Technical Report A-8. (University of Florida, Gainesville, 2008).

[65] Moulton EA (2011). Aversion-related circuitry in the cerebellum: responses to noxious heat and unpleasant images. J. Neurosci..

[66] Gear R (2013). Pain facilitation brain regions activated by nalbuphine are revealed by pharmacological fMRI. PLoS ONE.

[67] Worsley KJ, Evans AC, Marrett S, Neelin P (1992). A three-dimensional statistical analysis for CBF activation studies in human brain. J. Cereb. Blood. Flow. Metab..

[68] Kogler L (2015). Psychosocial versus physiological stress Meta-analyses on deactivations and activations of the neural correlates of stress reactions. Neuroimage.

[69] Smarr Karen L., Keefer Autumn L. (2011). Measures of depression and depressive symptoms: Beck Depression Inventory-II (BDI-II), Center for Epidemiologic Studies Depression Scale (CES-D), Geriatric Depression Scale (GDS), Hospital Anxiety and Depression Scale (HADS), and Patient Health Questionna. Arthritis Care & Research.

[70] Apkarian AV, Bushnell MC, Treede RD, Zubieta JK (2005). Human brain mechanisms of pain perception and regulation in health and disease. Eur. J. Pain..

[71] Namburi P, Al-Hasani R, Calhoon GG, Bruchas MR, Tye KM (2016). Architectural Representation of Valence in the Limbic System. Neuropsychopharmacology.

[72] Elman I, Borsook D, Lukas SE (2006). Food intake and reward mechanisms in patients with schizophrenia: implications for metabolic disturbances and treatment with second-generation antipsychotic agents. Neuropsychopharmacology.

[73] Kalivas PW, Volkow ND (2005). The neural basis of addiction: a pathology of motivation and choice. Am. J. Psychiatry.

[74] Geuze E (2007). Altered pain processing in veterans with posttraumatic stress disorder. Arch. Gen. Psychiatry.

[75] Strigo IA (2010). Neural correlates of altered pain response in women with posttraumatic stress disorder from intimate partner violence. Biol. Psychiatry.

[76] Defrin R (2008). Quantitative testing of pain perception in subjects with PTSD--implications for the mechanism of the coexistence between PTSD and chronic pain. Pain.

[77] Orr SP (2000). De novo conditioning in trauma-exposed individuals with and without posttraumatic stress disorder. J. Abnorm. Psychol..

[78] Kraus A (2009). Differentiation of pain ratings in combat-related posttraumatic stress disorder. Pain.

[79] Pitman RK, van der Kolk BA, Orr SP, Greenberg MS (1990). Naloxone-reversible analgesic response to combat-related stimuli in posttraumatic stress disorder. A pilot study. Arch. Gen. Psychiatry.

[80] Asmundson GJ, Katz J (2008). Understanding pain and posttraumatic stress disorder comorbidity: do pathological responses to trauma alter the perception of pain?. Pain.

[81] Ford GK, Finn DP (2008). Clinical correlates of stress-induced analgesia: evidence from pharmacological studies. Pain.

[82] Flack WF, Litz BT, Hsieh FY, Kaloupek DG, Keane TM (2000). Predictors of emotional numbing, revisited: a replication and extension. J. Trauma. Stress.

[83] Litz BT, Gray MJ (2002). Emotional numbing in posttraumatic stress disorder: current and future research directions. Aust. N. Z. J. Psychiatry.

[84] Saga Y, Hoshi E, Tremblay L (2017). Roles of Multiple Globus Pallidus Territories of Monkeys and Humans in Motivation, Cognition and Action: An Anatomical, Physiological and Pathophysiological Review. Front. Neuroanat..

[85] Deisseroth K (2015). Optogenetics: 10 years of microbial opsins in neuroscience. Nat. Neurosci..

[86] Kim CK, Adhikari A, Deisseroth K (2017). Integration of optogenetics with complementary methodologies in systems neuroscience. Nat. Rev. Neurosci..

[87] Cromwell HC, Mears RP, Wan L, Boutros NN (2008). Sensory gating: a translational effort from basic to clinical science. Clin. Eeg. Neurosci..

[88] Wang AL, Mouraux A, Liang M, Iannetti GD (2010). Stimulus novelty, and not neural refractoriness, explains the repetition suppression of laser-evoked potentials. J. Neurophysiol..

[89] Elman, I., Borsook, D. Threat Response System: Parallel Brain Processes in Pain vis-à-vis Fear and Anxiety. *Front. Psychiatry* eCollection (2018). 10.3389/fpsyt.2018.00029 PMC5826179 29515465

[90] Neylan TC (1999). Sensory gating in chronic posttraumatic stress disorder: reduced auditory P50 suppression in combat veterans. Biol. Psychiatry.

[91] Karl A, Malta LS, Maercker A (2006). Meta-analytic review of event-related potential studies in post-traumatic stress disorder. Biol. Psychol..

[92] Wilson TW, Heinrichs-Graham E, Proskovec AL, McDermott TJ (2016). Neuroimaging with magnetoencephalography: A dynamic view of brain pathophysiology. Transl. Res..

[93] Elman I, Borsook D, Volkow ND (2013). Pain and suicidality: insights from reward and addiction neuroscience. Prog. Neurobiol..

[94] Posner J, Russell JA, Peterson BS (2005). The circumplex model of affect: an integrative approach to affective neuroscience, cognitive development, and psychopathology. Dev. Psychopathol..

[95] Crombez G, Eccleston C, Baeyens F, Eelen P (1998). When somatic information threatens, catastrophic thinking enhances attentional interference. Pain.

[96] Ploghaus A (2001). Exacerbation of pain by anxiety is associated with activity in a hippocampal network. J. Neurosci..

[97] Sharp TJ, Harvey AG (2001). Chronic pain and posttraumatic stress disorder: mutual maintenance?. Clin. Psychol. Rev..

[98] Asmundson GJ, Katz J (2009). Understanding the co-occurrence of anxiety disorders and chronic pain: state-of-the-art. Depress Anxiety.

[99] Asmundson GJ, Coons MJ, Taylor S, Katz J (2002). PTSD and the experience of pain: research and clinical implications of shared vulnerability and mutual maintenance models. Can. J. Psychiatry.

[100] Liedl A, Knaevelsrud C (2008). Chronic pain and PTSD: the Perpetual Avoidance Model and its treatment implications. Torture.: Q. J. Rehabil. Torture. Vict. Prev. Torture..

[101] Liedl A (2010). Support for the mutual maintenance of pain and post-traumatic stress disorder symptoms. Psychol. Med..

[102] Sloan DM, Marx BP, Lee DJ, Resick PA (2018). A Brief Exposure-Based Treatment vs Cognitive Processing Therapy for Posttraumatic Stress Disorder: A Randomized Noninferiority Clinical Trial. JAMA Psychiatry.

[103] Glover H (1993). A preliminary trial of nalmefene for the treatment of emotional numbing in combat veterans with post-traumatic stress disorder. Isr. J. Psychiatry Relat. Sci..

[104] Baker DG (1997). Cerebrospinal fluid and plasma beta-endorphin in combat veterans with post-traumatic stress disorder. Psychoneuroendocrinology.

[105] Hamner MB, Hitri A (1992). Plasma beta-endorphin levels in post-traumatic stress disorder: a preliminary report on response to exercise-induced stress. J. Neuropsychiatry Clin. Neurosci..

[106] Hoffman L, Burges Watson P, Wilson G, Montgomery J (1989). Low plasma beta-endorphin in post-traumatic stress disorder. Aust. N. Z. J. Psychiatry.

[107] De Felice M, Porreca F (2009). Opiate-induced persistent pronociceptive trigeminal neural adaptations: potential relevance to opiate-induced medication overuse headache. Cephalalgia: Int. J. Headache.

[108] Okada-Ogawa A, Porreca F, Meng ID (2009). Sustained morphine-induced sensitization and loss of diffuse noxious inhibitory controls in dura-sensitive medullary dorsal horn neurons. J. Neurosci..

[109] Coderre TJ, Katz J, Vaccarino AL, Melzack R (1993). Contribution of central neuroplasticity to pathological pain: review of clinical and experimental evidence. Pain.

[110] Mao J, Price DD, Mayer DJ (1995). Mechanisms of hyperalgesia and morphine tolerance: a current view of their possible interactions. Pain.

[111] Mao J, Sung B, Ji RR, Lim G (2002). Chronic morphine induces downregulation of spinal glutamate transporters: implications in morphine tolerance and abnormal pain sensitivity. J. Neurosci..

[112] Norman SB, Stein MB, Dimsdale JE, Hoyt DB (2008). Pain in the aftermath of trauma is a risk factor for post-traumatic stress disorder. Psychol. Med..

[113] Bryant RA, Creamer M, O’Donnell M, Silove D, McFarlane AC (2009). A study of the protective function of acute morphine administration on subsequent posttraumatic stress disorder. Biol. Psychiatry.

[114] Holbrook TL, Galarneau MR, Dye JL, Quinn K, Dougherty AL (2010). Morphine use after combat injury in Iraq and post-traumatic stress disorder. N. Engl. J. Med..

[115] McGhee LL, Maani CV, Garza TH, Gaylord KM, Black IH (2008). The correlation between ketamine and posttraumatic stress disorder in burned service members. J. Trauma.

[116] Ibarra P (1994). An unusual reaction to opioid blockade with naltrexone in a case of post-traumatic stress disorder. J. Trauma. Stress.

[117] Liberzon I (2007). Altered central micro-opioid receptor binding after psychological trauma. Biol. Psychiatry.

[118] Bills LJ, Kreisler K (1993). Treatment of flashbacks with naltrexone. Am. J. Psychiatry.

[119] Lubin G, Weizman A, Shmushkevitz M, Valevski A (2002). Short-term treatment of post-traumatic stress disorder with naltrexone: an open-label preliminary study. Hum. Psychopharmacol..

[120] Kozaric-Kovacic D (2009). Pharmacotherapy treatment of PTSD and comorbid disorders. Psychiatr. Danub..

[121] Casey KL (1999). Forebrain mechanisms of nociception and pain: analysis through imaging. Proc. Natl Acad. Sci. USA.

[122] Elman I (1998). Effect of acute metabolic stress on pituitary-adrenal axis activation in patients with schizophrenia. Am. J. Psychiatry.

[123] Elman I (2012). Yohimbine-induced amygdala activation in pathological gamblers: a pilot study. PLoS ONE.

[124] Cloninger CR, Svrakic DM, Przybeck TR (1993). A psychobiological model of temperament and character. Arch. Gen. Psychiatry.

[125] Moeller-Bertram T (2014). Evidence for acute central sensitization to prolonged experimental pain in posttraumatic stress disorder. Pain. Med..

